# Diabetes and Its Cardiovascular Complications: Comprehensive Network and Systematic Analyses

**DOI:** 10.3389/fcvm.2022.841928

**Published:** 2022-02-17

**Authors:** Hao Wu, Vikram Norton, Kui Cui, Bo Zhu, Sudarshan Bhattacharjee, Yao Wei Lu, Beibei Wang, Dan Shan, Scott Wong, Yunzhou Dong, Siu-Lung Chan, Douglas Cowan, Jian Xu, Diane R. Bielenberg, Changcheng Zhou, Hong Chen

**Affiliations:** ^1^Department of Surgery, Vascular Biology Program, Harvard Medical School, Boston Children's Hospital, Boston, MA, United States; ^2^Department of Medicine, Harold Hamm Diabetes Center, University of Oklahoma Health Sciences Center, Oklahoma, OK, United States; ^3^Division of Biomedical Sciences, School of Medicine, University of California, Riverside, Riverside, CA, United States

**Keywords:** diabetes, comprehensive network, system analysis, cardiovascular disease complications, peripheral artery disease

## Abstract

Diabetes mellitus is a worldwide health problem that usually comes with severe complications. There is no cure for diabetes yet and the threat of these complications is what keeps researchers investigating mechanisms and treatments for diabetes mellitus. Due to advancements in genomics, epigenomics, proteomics, and single-cell multiomics research, considerable progress has been made toward understanding the mechanisms of diabetes mellitus. In addition, investigation of the association between diabetes and other physiological systems revealed potentially novel pathways and targets involved in the initiation and progress of diabetes. This review focuses on current advancements in studying the mechanisms of diabetes by using genomic, epigenomic, proteomic, and single-cell multiomic analysis methods. It will also focus on recent findings pertaining to the relationship between diabetes and other biological processes, and new findings on the contribution of diabetes to several pathological conditions.

## Introduction

Diabetes mellitus is a critical public health issue that causes incapacitation and mortality in both acute and chronic complications of the disease. It affects various races and populations. The prevalence of diabetes in adults globally was ~6.4% in 2010 and was predicted to rise to 7.7% in 2030 (1). Diabetes in general is a chronic metabolic disease, characterized by β-cell dysfunction and/or insulin resistance and hyperglycemia.

Diabetes mellitus is classified as a spectrum of metabolic disorders in which the American Diabetes Association (ADA) divides into four categories: type 1 diabetes (T1D), type 2 diabetes (T2D), monogenic diabetes (MD) and gestational diabetes (GD). T1D is an autoimmune illness that is caused by the beta cells of the pancreas' Langerhans islets being destroyed. These beta cells secrete insulin, and thus insulin has to be used throughout T1D patient lives. T1D accounts for around 5–10% of all diabetic cases. Insulin resistance and impaired secretion, as well as increased hepatic glucose synthesis, are all pathological symptoms of T2D. Approximately 90% of diabetics have T2D (2). In fact, over 29 million people in the US have T2D. Many risk factors, both genetic and non-genetic, have been identified that play a role in the process of T2D. For example: obesity, physical inactivity, advanced age, hypertension, hyperlipidemia, and family history are all risk factors. Furthermore, cardiovascular disease, stroke, periodontal disease, neuropathy, retinopathy, foot ulcers, and amputations are well studied complications associated with T2D (3).

Monogenic diabetes is caused by a defect in a single gene and often has a similar clinical presentation to T1D and T2D. Gestational diabetes was once considered to be an early stage of T2D (3). Now it is thought that there is increased susceptibility to T2D enabled by pregnancy-induced insulin resistance which is characteristic of gestational diabetes. After the patient has given birth, typically their glucose levels will return to normal.

The significant health consequences of diabetes have led to an emphasis on early identification, management and treatment strategies for diabetic patients. In this review, we will summarize recent findings on the mechanisms of diabetes that have used genomic, epigenomic, proteomic, and multiomics single-cell analysis methods (4, 5). Given the huge contribution of cardiovascular complications to the severity in outcome of diabetes, we will also discuss recent findings on the relationship between diabetes and the physiological systems it affects, such as lymphangiogenesis, angiogenesis, gut microbiota diversity, and more. With further research being done in these areas, we will be better equipped to therapeutically intervene in the development of diabetes and its associated cardiovascular complications.

## Advancement in Elucidating the Mechanisms of Diabetes

### Genomics Research

Genomic analysis to detect risks for chronic diseases such as diabetes is quickly progressing in the clinical setting, thanks to the use of next-generation sequencing technology including whole-genome sequencing.

T1D is a multifaceted disorder with genetic and environmental risk factors. In the last several decades, numerous studies have been conducted to identify T1D-susceptibility genes in which more than 40 different genetic loci associated with T1D have been identified (6, 7). The human leukocyte antigen (HLA) area on chromosome 6p21, protein tyrosine phosphatase non-receptor type 22 (PTPN22) on 1p13, interleukin 2 receptor subunit alpha (IL2RA) on 10p15, the insulin gene (INS-VNTR) locus on 11p15, as well as the cytotoxic T-lymphocyte associated protein 4 (CTLA4) locus on 2q33 are all among the different genetic loci associated with T1D (8). CTLA4 is an immunoglobulin that plays an important role in the pathogenesis of autoimmune disorders like T1D (9). The interleukin-2 receptor complex's -chain is encoded by the IL2RA gene, which has eight exons. In regulatory T-cells, the expression of IL2RA is essential in controlling the immune response and preventing autoimmune disease (10). Recently, several studies have been conducted and found the frequency of them in different populations to be very different (11, 12).

T2D is a complex disease that leads to serious consequences. Thus, there has been an emphasis on early identification of individuals at high risk for T2D. Several clinical factors correlated with T2D risk that can be identified early on include body mass index (BMI), age, and family history. With the advancement in genomics, including genomic factors in risk assessment and management could make risk prediction and treatment of T2D more precise.

Around 40% of the risk, onset, and progression of diabetes is due to genetic factors, which varies from person to person (13). There have been more than 50 loci associated with T2D risk identified by the Genome Wide Association Studies (GWAS) since 2007 (14–17). Several genes associated in insulin production, glucose metabolism, and beta-cell activity have been identified. One study (18) found the association of 21 genetic variants with T2D and confirmed that individuals with a high genetic score had an increased risk of T2D. The study (18) looked at 65 single nucleotide polymorphisms (SNPs), seven of which were found in four genes which are Gli-similar 3 (GLIS3), transcription factor-7–like 2 (TCF7L2), leucine rich repeat containing G protein-coupled receptor 5 (LGR5), and protein tyrosine phosphatase receptor type D (PTPRD). These 7 SNPs were strongly associated with T2D. GLIS3 is a diabetes susceptibility gene that participates in the propagation of pancreatic beta cells. TCF7L2 was observed to have a relationship with BMI and has been demonstrated to affect β-cell responsiveness to insulin.

Furthermore, because oral anti-hyperglycemic drugs are affected by pharmacogenomic variation in a high number of T2D patients, research suggests genomics could play a role in choosing the most successful therapy (19). Several studies have revealed that genetic variations are involved in drug absorption, transport, metabolism, and action, and that these variations may alter drug pharmacokinetics or pharmacodynamics (20, 21). Since the susceptibility loci, identified by GWAS, for T2D mellitus alter insulin secretion and/or sensitivity, they may also influence the efficacy of the insulin secretagogue and/or sensitizer. The potassium voltage-gated channel subfamily Q member 1 (KCNQ1) gene, for example, has been linked to repaglinide and rosiglitazone efficacy in East Asians and at the same time confers the highest risk of T2D Mellitus in East Asians (22, 23).

In overall, existing knowledge of the role of genetic variables in diabetes supports the notion that diabetes is a complicated disease that differs from person to person. In addition, important information on the genetic underpinnings for various therapeutic responses to pharmacologic therapy is now being discovered. With increasing knowledge about the importance of genetic information in the onset, progression and treatment of diabetes, genome-based strategies can be considered to improve the risk prediction and customized management of individual patients. Both of which will contribute to better health outcomes for diabetic patients.

### Epigenetics Research

In the past few years it has been determined that mainly environmental factors have been considered as predisposing factors for weight gain or the development of T2D (24). Despite significant efforts to find genetic susceptibility variations, little progress has been made, and the common genetic variables that cause diabetes susceptibility can only account for a small portion of individual risk variants. In addition, there is evidence that the current diabetes epidemic is driven by environmental factors. Recent studies have shown that in addition to a good balance between energy intake and energy expenditure, normal metabolic regulation in adulthood is also affected by the pre- and post-natal environment. In fact, maternal calorie restriction during pregnancy can alter the metabolic phenotype of their children by epigenetic control of certain genes, which can be passed down to future generations. Thus, it is important to identify the epigenetic markers of diabetes and the methylation and/or histone acetylation levels of genes involved in metabolic processes. Recent studies have pointed out that endocrine disruptors, which are chemicals that interfere with many homeostatic mechanisms, play a role in the high incidence of diabetes. Given the existing data on the effects of endocrine disruptors such as obesogens, it seems that exposure to these disruptors may play an important role in the diabetes pandemic (24).

**Figure 1:**
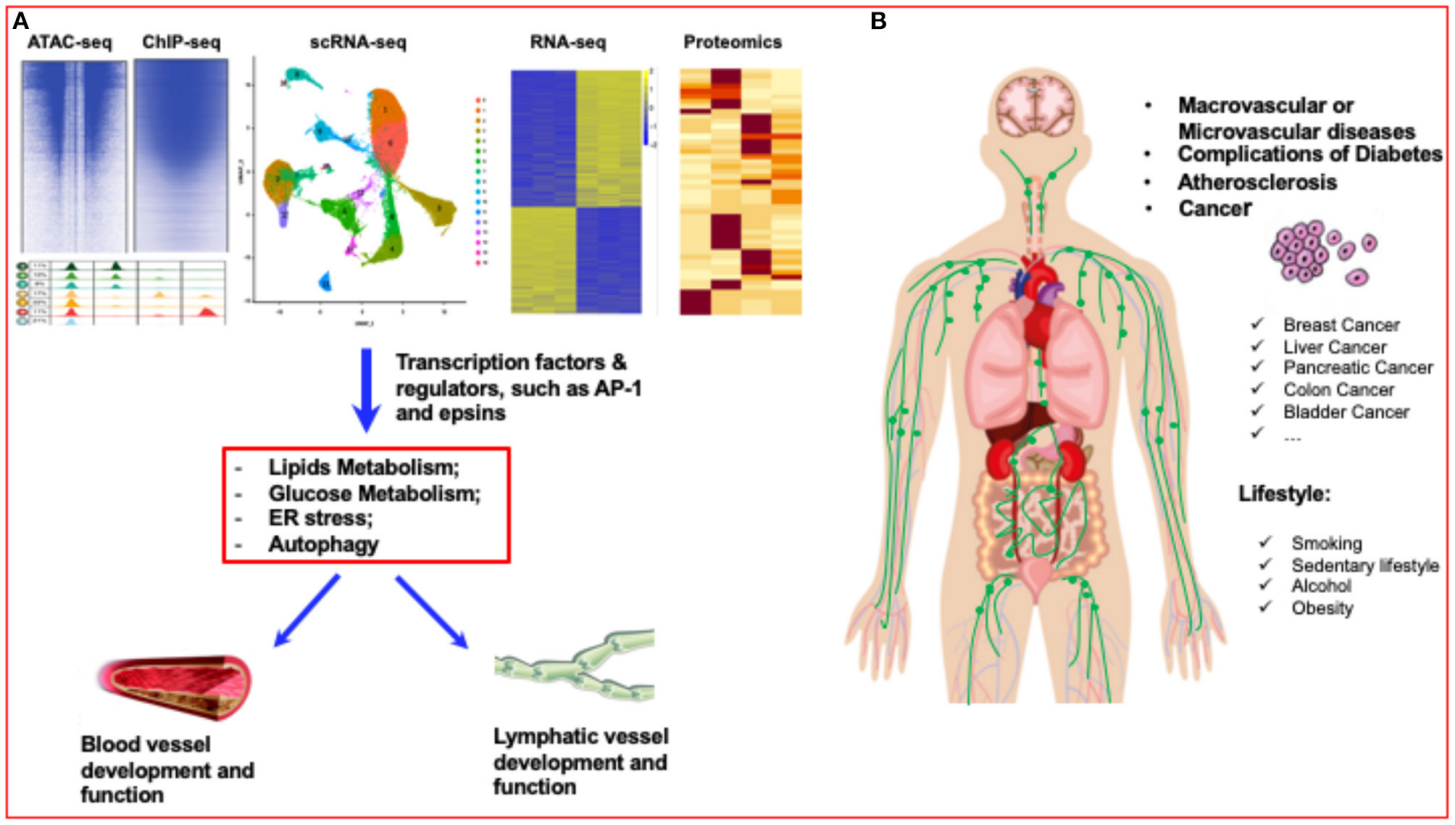
Diabetes research has entered a new era of single-cell biology. **(A)** Single-cell analysis has entered the multiomics age. By using multiomics single-cell analysis, such as ATAC-seq, ChIP-seq, scRNA-seq, RNA-seq and proteomics analysis, the transcriptome factors or regulators of blood vessels and lymphatic vessels can be accurately identified. **(B)** Lifestyles, such as smoking, sedentary lifestyle, alcohol, and obesity can significantly affect the vascular diseases, such as atherosclerosis, diabetes, even including different cancers.

Epigenetics has been defined as a heritable change in gene function without changes in the nucleotide sequence, however this is not a universal definition (25). Epigenetic changes can be handed down from one cell generation to the next (mitotic inheritance) as well as between generations of species (meiotic inheritance). Epigenetics can be affected by the environment, which makes it a potentially important pathogenic mechanism for complex multifactorial diseases such as T2D (Figure 1). DNA methylation, histone modification, and microRNA are all epigenetic factors that can help explain how cells with the same DNA differentiate into different cell types with different phenotypes (26), all of which aid in explaining how cells with identical DNA differentiate into different cell types with different phenotypes. DNA methylation and histone modification, in particular, are important in the pathogenesis of T2D.

DNA methylation necessitates the activity of methyltransferases, of which there are two types: DNA methyltransferase 1 (DNMT1), which replicates the DNA methylation pattern (maintains methylation) between cell generations during replication, and DNA methyltransferase 3A (DNMT3a) and DNA methyltransferase 3 beta (DNMT3b), which are both responsible for DNA de novo methylation (27). The way in which this DNA methylation occurs is still poorly understood and needs further research if we are to understand the mechanisms behind the pathogenesis of T2D. For recent research on DNA methylation, see Patra et al. (28). Examples of ways to determine these genetic signals include using the Chromatin analysis methods, such as ATAC-seq and DNase-seq, which have been applied to a large number of islets to generate aggregated spectra that mask important cells and regulate heterogeneity (29, 30). In addition, GWAS have been able to identify >400 independent signals that encode genetic predispositions for T2D (31). Finally, more than 90% of linked SNPs are found in non-coding regions and contain chromatin-defined islet enhancer elements, indicating the presence of significant transcriptional regulatory components for diabetes disease risk (32).

Histone modification starts with the formation of chromatin. The nucleosome, which consists of around 147 DNA base pairs surrounding histone octamers, is the most fundamental component of chromatin. Histone octamers are composed of H3-H4 tetramers, with one H2A-H2B dimer on each side. Although the core histones are densely packed, histone modifying enzymes can alter their NH2 terminal tails, causing acetylation, methylation, phosphorylation, SUMO acylation, or ubiquitination (33). An example of this modification involves histone modifying enzyme HDAC which has been shown to remove histone acetyltransferase (HAT) and add acetyl groups (33–35) to lysine residues in the tail of histones. Although enhanced HAT activity and histone acetylation have been linked to increased gene transcription, the exact mechanism that promotes transcription is unknown (36). On top of this, histone methyltransferases and histone demethylases have been shown to mediate HAT activity (37). Taken together, understanding how histone modification and acetylation are regulated is important for determining the transcription mechanism's access to DNA, as well as DNA replication, recombination, and chromosomal organization, all of which are crucial in understanding its relationship to T2D.

### Proteomics Research

Integrative profiling of proteins expressed in cells, tissues, and organs has been done using proteomics. Proteomics research has provided potential tools for the systematic investigation of proteins that are differently expressed between healthy individuals and cancer patients (38), as well as Alzheimer's disease (39) and diabetes patients (40). Proteomics has been widely used in diabetes studies focusing on different stages of diabetes with diverse sample sources.

A longitudinal study of the human plasma proteome discovered possible protein indicators in T1D progression, resulting in a promising list of protein markers that dysregulate temporally before islet autoimmunity develops. (41). Key enzymes against oxidative stress, CAT and SOD1, were identified (41). Eri Takahashi et al. (42) carried out serum proteomics using a T2D mouse model and identified differentially expressed proteins in the prediabetic state, among which the level of serine protease inhibitor (SERPIN)A3 was found to be elevated significantly. This change was also confirmed to be increased in T2D patients, indicating SERPINA3 could be used for the early detection of type 2 diabetes mellitus (42).

Proteomic analysis of human islets from patients with T1D was also carried out (43). Upon human pancreatic islets being exposed to palmitate, lipidomics and proteomics were done which revealed proteins implicated in the action of saturated fatty acids as well as potential pathways for how chronic saturated free fatty acids disrupt beta-cells and lead to the development of T2D mellitus (44).

Adipose tissue is an endocrine organ secreting multiple bioactive factors such as leptin, tumor necrosis factor-α and interleukin-6, all of which influence insulin resistance and β-cell dysfunction (45). White adipose tissue (WAT) and brown adipose tissue (BAT) are two types of adipose tissue that are linked to the development of metabolic diseases. As a result of these studies, differentially expressed proteins involved in cytoskeleton function and structure, oxidative stress, inflammation, and retinoid metabolism have been identified in TD-related adipose tissues (46–48).

Proteomic analysis of protein expression in diabetic patient samples provides detailed qualitative and quantitative information on the proteins implicated in the course of diabetes. This could potentially yield pathomechanistic insights and lead to the development of new therapeutic targets for diabetes intervention. The importance of such promising potential markers warrants greater investigation and research.

### Single-Cell Multiomic Analysis

Characterizing the transcriptome profile of a single cell through single-cell RNA sequencing (scRNA-Seq) has become a universal tool for identifying known and new cell types, as well as understanding tissue structure and function, ushering in a new era of single-cell biology (4, 5, 49) (Figure 1). This has been shown to be especially true in complex organs and tissues with a high degree of cellular heterogeneity, such as mammalian brains and tumors (50, 51).

In the past few years, using scRNA-Seq to analyze pancreas cells at the individual level has made great strides. Among them, exciting discoveries have been made in the immunology of T1D and T2D. For example, scRNA-Seq analysis has shown that increased expression of the anion transporter SLC26A9 delayed the onset of cystic fibrosis diabetes, a unique type of diabetes that has similar characteristics to T1D and T2D (52).

ScRNA-seq has also been shown to be useful in the cellular characteristics of human *in vitro* β cell differentiation, providing a perspective for the use of human stem cell differentiation as a useful therapeutic that could guide future efforts to focus on islet cell differentiation and regeneration in diabetic models (53). The single-cell transcriptome analysis of the human ductal tree indicates that progenitors might be activated *in situ* for therapeutic purposes (54). Other successful examples include a study done by Baron and Muraro et al. who used scRNA-Seq to deconvolute a large number of human and mouse pancreas gene expression samples to detect disease-related differential expression. The data set provided resources for discovering new cell type-specific transcription factors, signal receptors, and medical-related genes in human and mouse pancreases (55, 56). Another research team conducted single-cell transcriptomics analysis of the human endocrine pancreas and also demonstrated the powerful functions of single-cell RNA-seq (57).

**Figure 2:**
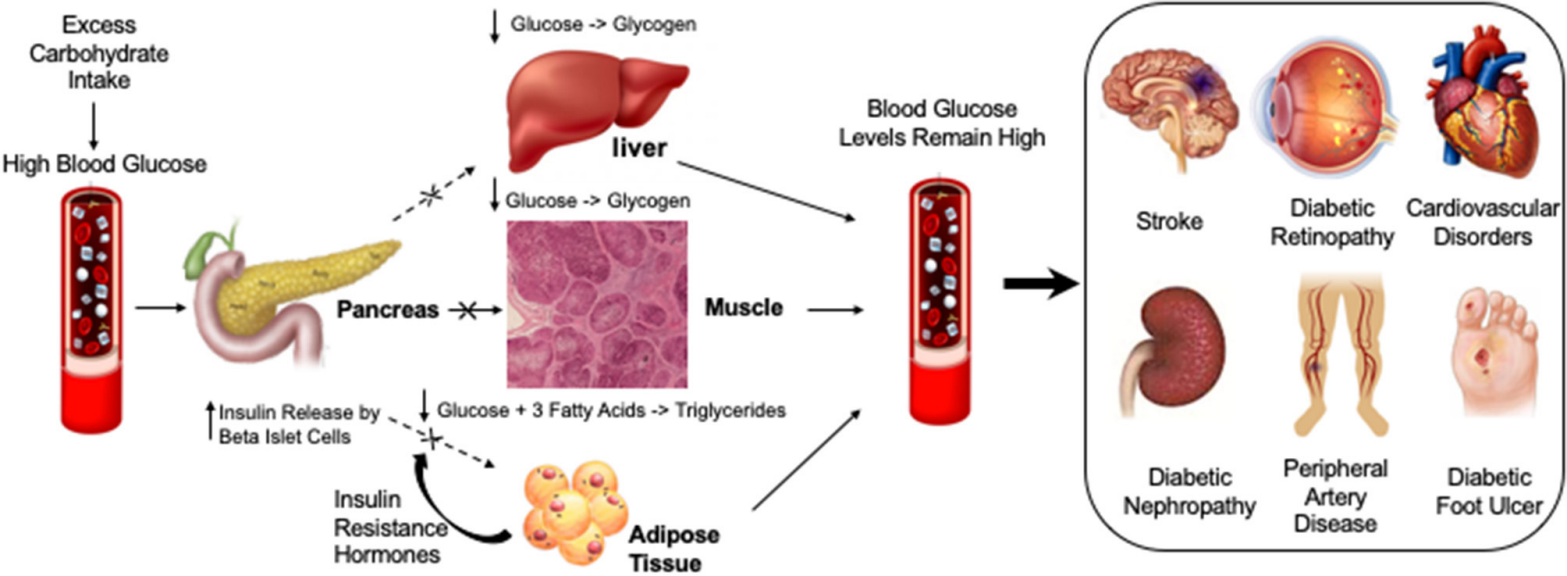
Diabetic compilations are caused by insulin resistance leading to persistently elevated glucose levels or hyperglycemia. Different factors that may cause or exacerbate blood glucose levels are highlighted under “Lifestyle.” High levels of blood glucose in diabetic vessels can cause different diabetic complications such as Diabetic Foot Ulcer, Diabetic Nephropathy, Cardiovascular Disorders, Diabetic Retinopathy, Peripheral artery disease and Stroke.

T2D is a complex disease characterized by pancreatic islet dysfunction, insulin resistance, and blood sugar level disturbances (Figure 2). Pancreatic islets are a mixture of cell types expressing different hormonal programs, so each cell type may contribute differently to the underlying regulatory processes that regulate T2D-related transcription circuits. There are many ways to use genetic signals of T2D to identify the type of activity undergone by islet cells and to provide higher-resolution mechanical insights into genetically encoded risk pathways for T2D. Single-cell genomics has exploded in popularity during the last decade. The most prevalent technique is single-cell RNA sequencing (RNA-seq), which assesses gene expression. Other approaches examine methylation, genetic variation, protein abundance, and chromatin accessibility, among other things. To date, single-cell analysis has entered the multiomics age (4, 5). Some research has combined these methodologies—and the associated layers of data—with “multiomics” investigations. In a technique called scNMT-seq, Argelaguet integrated gene expression profiling, methylation, and chromatin accessibility. Another technique called CITE-seq profiles both transcription and protein abundance. An additional technique known as G&T-seq captures both genomic DNA and RNA (5, 58, 59). A recent multiomics single-cell analysis identifies new cell types and processes that may contribute to the pathogenesis of T1D immunity as well as provide new cellular and molecular insights into human pancreatic function (bioRxiv, 2021, doi: 10.1101/2021.01.28.42859). In addition, due to the rapidly growing suite of software tools, there will be more and more applications of multiomics single-cell analysis in diabetes research (Figure 1).

While genomic analysis on diabetic risk prediction and pharmacological responses in the clinic suggests the importance for the development of individual/personal-based diabetic medicine, epigenetics research has generated new knowledge about one of the most important environmental risk factors for diabetes. Advances in proteomics research and single-cell multiomic analysis have been providing unpreceded insights into specific cell-type and molecular networks involved in the pathogenesis of diabetes. In overall, mechanistic findings in these areas will help to better understand the mechanisms of diabetes, which would lead to identifying new therapeutic targets in the pathophysiological systems that cause diabetic complications.

## Diabetes and Cardiovascular Systems and Beyond

### Angiogenesis and Diabetes

Angiogenesis is a well-studied process that entails the formation of new blood vessels from existing blood vessels and is involved in a large number of physiological and pathological conditions. During embryonic development, wound healing, menstruation, and angiogenesis must occur to provide adequate blood flow and oxygenation to growing tissues (60). Vascular disease associated with aberrant angiogenesis is a feature of some long-term diabetic consequences. Diabetic retinopathy and nephropathy are both linked to excessive angiogenesis. Inhibition of angiogenesis can lead to impaired wound healing, impaired development of coronary collateral vessels, embryonic vascular disease in pregnancy with maternal diabetes, and transplant rejection in diabetic recipients (60).

The majority of the vasculature in a healthy adult is dormant, with only 0.01 percent of endothelial cells undergoing division. Excessive or insufficient vascular growth as in the case of pathological angiogenesis contributes to numerous non-neoplastic disorders. In some diseases, vessels do not grow, but rather abnormally remodel (61). Angiogenesis has been recognized as a hallmark of cancer and various metabolic and inflammatory diseases, such as obesity, T2D, atherosclerosis and NAFLD (61). Both physiological and pathological angiogenic variants are controlled by carefully orchestrated, temporally and spatially controlled signals from surrounding tissues, and it is the sum of these signals that causes the sequential release of angiogenic stimulators (e.g., VEGF, bFGF, PDGF) and inhibitors (e.g., endostatin, angiostatin, thrombospondin). In the past decade, research in molecular mechanisms underlining pathological angiogenesis (blood vessel growth) has grown at an explosive rate, and has led to the approval of anti-angiogenic drugs for the treatment of cancer and eye diseases (62).

Endothelial progenitor cells (EPCs) are a subtype of progenitor cells, which are first isolated in the circulation (63), and have the capacity to differentiate into mature ECs *in vitro* and *in vivo* (64). Dysfunctional EPCs with impaired vascular repairing capacity have been reported in diabetes (65). In a small clinical study of cardiovascular disease patients with or without diabetes, an increase in EPC numbers was promoted by statin administration, which was associated with HDL changes (66). Although large clinical trials are needed to validate EPCs as independent indicators of cardiovascular risk (67), several recent pre-clinical studies support its role in restoring angiogenesis in diabetes. For example, EC-specific overexpression of metallothionein (MT), an antioxidant protein, prevented impairment of angiogenesis in a hind limb Ischemia model of mice fed a high-fat diet (HFD) or treated with streptozotocin (STZ) (68). The protection was likely due to the preserved function of EPC, attributable to a reduction in oxidative stress and an enhanced expression of hypoxia-inducible factor 1a (HIF-1a), stromal cell–derived factor (SDF-1), and VEGF in ischemic tissues (68). Endothelial-colony-forming cells (ECFCs) are isolated as a novel type of progenitor cells (69). Like EPCs, ECFCs have the potential capacity to promote angiogenesis *in vitro* and *in vivo* which could be impaired by diabetes with similar mechanisms (70). ECFCs, which are of endothelial origin, are believed to be a better cell therapy tool for vascular regeneration in ischaemic models (70) because ECFCs express CD31^+^, CD34^+^, CD146^+^, VEGFR2^+^, and von Willebrands factor (69). On the other hand, EPCs are of myeloid origin and express CD31^+^, CD34^+^, CD45^+^, VEGFR2^+^, and Tie-2^+^, with a low proliferative capacity. Further investigation, however, needs to be done to prove the applicability of ECFCs in pre-clinical and clinical settings (70).

Although diabetes can cause a variety of pathologies, vascular complications account for most of the morbidity and mortality of diabetes (71). Furthermore cardiovascular disease causes 75% of the deaths of diabetic patients (71). Diabetes can cause macrovascular and microvascular problems characterized by endothelial dysfunction, which can have serious consequences for wound healing (72, 73). Inhibition of the vascular endothelial growth factor (VEGF-VEGFR2) signal axis is related to endothelial dysfunction typical of diabetes (74, 75). Under high glucose exposure, VEGFR2 ligand and intrinsic kinase-independent phosphorylation occurs in the Golgi apparatus of endothelial cells, thereby impairing transport of receptors to the cell surface. The result is that VEGFR2 on the plasma membrane of endothelial cells gradually decreases, thereby weakening the angiogenic response of diabetes.

There is evidence that beta cells are an important ally of islet endothelial cells (EC). In addition, ECs seem to directly affect the expression and secretion of insulin genes and the survival of β cells. Pancreatic islet EC is an important partner for β cell function (76). This dynamic relationship is very important in the context of type 1 and type 2 diabetes and has been shown to establish the potential of EC or its progenitor cells to enhance the reconstitution of blood glucose control after islet transplantation in animal models (76, 77). Dysfunctional islet endothelium may lead to the progression of type 1 diabetes, the deterioration of type 2 diabetes, and the failure of islet transplantation (76). Treatments that prevent the breakdown of the complex β-cell/EC axis in pancreatic islets or restore this crosstalk may improve the prognosis of diabetic patients in the future.

In order to assess pathological or diabetic angiogenesis, there are many *in vivo, ex vivo*, and *in vitro* bioassays that are available for proper evaluation of angiogenesis (78). *In vitro* bioassays are used to detect EC cells proliferation, tube formation and migration, and *in vivo* bioassays, such as the corneal micropocket assay, matrigel plug assay, tail edema, and dermal punch biopsy wound healing assays are all used to evaluate angiogenesis and lymphangiogensis (78). Abnormal angiogenesis contributes to vascular disorders in diabetes. Provided the distinct role of angiogenesis in macrovascular and microvascular complications of diabetes, future studies should identify tissue-specific regulators of angiogenesis and their underlying mechanisms by using conventional approaches coupled with single-cell multiomic analysis and other integrative methodologies.

### Lymphangiogenesis and Diabetes

Lymphatic vessels and blood vessels create an intricate system that aids in the management of tissue pressure and the production of edema. Lymphatic endothelial cells and lymphangiogenesis play critical roles of homeostasis, metabolism and immunity in both physiological and pathological angiogenesis. Except for the reproductive organs during ovarian cycles and pregnancy, the majority of lymphatic vessels in adult tissues are dormant (79). A variety of pathological conditions such as inflammation and tumor formation promote lymphangiogenesis and lymphatic vessel remodeling in the adult (80). In addition, lymphangiogenesis is enhanced post organ transplantation for inducing immune system reactivation in the draining lymph node, resulting in organ rejection (81). In general, a consequence of chronic complicated disorders, such as diabetes, is poor lymphangiogenesis (82) (Figure 1).

Recently, studies have suggested the therapeutic roles of lymphangiogenesis in various pathological conditions. For example, excess lymphangiogenesis favors metastasis and inflammation, however insufficient lymphangiogenesis can cause lymphedema (82, 83). The reasons and effects of adult lymphangiogenesis is still up for debate for whether it is beneficial or detrimental. Enhancing lymphangiogenesis protects against diabetes and other metabolic diseases (82). Obesity and diabetes have been linked to a lack of lymphatic architecture and impaired lymphatic function (84–89). Diet-induced obesity impairs lymphangiogenesis as indicated by decreased LYVE1 positive lymphatic vessel density (87), and corroborating impaired lymphangiogenesis in diabetic mice (82, 90). Transcription factor prospero homebox 1 (Prox1) is one of the key regulators of lymphangiogensis (46). Disturbed lymphangiogenesis in *Prox1*^+/−^ mice induced obesity, coupled with decreased lymph flow in adult mouse models (91, 92). Obesity is considered to make up 80–85% of the risk of developing T2D. Recent studies have shown that obese people are 80 times more likely to develop T2D than people with a BMI below 22 (https://www.diabetes.co.uk/diabetes-and-obesity.html). Enhancing VEGFR3 expression and VEGFR3 signaling by depleting epsins modulates VEGFR2/3 in endothelial cells promotes lymphangiogenesis and augments lymph flow in type 2 diabetic mice (82, 93) (Figure 1). LEC specific epsin depletion increased VEGFR3 expression and reduced VEGFR3 endocytosis and degradation resulting in enhanced wound-healing and surgery-induced lymphedema resolutions in diabetic mice (82). Since the animal model was in stage III of diabetic progression via STZ injection combined with HFD, the promoting lymphangiogenesis in the diabetic mouse did not increase insulin sensitivity. This might be due to damaged pancreatic β-cells having no response to any excess glucose which would usually decrease insulin responsiveness. In HFD-induced obese mice, overexpressing VEGF-D increases lymphatic density in adipose tissue, which lowers local immune cell buildup and improves systemic metabolic response by reducing insulin resistance and enhancing insulin sensitivity (94). There are little lymphatic vessels in mice white adipose tissue and promoting *de novo* lymphangiogenesis in adipose tissue enhanced insulin sensitivity in HFD mice treated for 16 weeks. Even if the body weight was similar between adipose tissue-VEGF-D-overexpressed mice and littermate controls, enhanced lymphangiogenesis in adipose tissue increased insulin sensitivity and reduced insulin resistance compared to the controls. Lymphatic vessels play a role in glycerol clearance and removal of infiltrated immune cells which improves metabolism in obese mice (94). Different fat pads develop and mature at different rates (95). For example, epididymal adipose tissue exhibits very little lipid component until P4 while other fat pads (e.g., subcutaneous, retroperitoneal adipose tissue) display high lipid component on postnatal day 1 (95). VEGFR3 and Prox1 show a significant percentage of distribution in the epididymal adipose tissue until postnatal day 5, indicating lymphangiogenesis may play an inhibitory role in lipid deposition in adipose tissues. Notably, angiogenesis contributes to adipose tissue development (95) while lymphatics might play an inhibitory role in lipid deposition in the adipose tissue. While VEGF-D also promotes angiogenesis via VEGFR2 (96), there is no significant increase in angiogenesis by VEGF-D overexpression in adipose tissues (94, 97). Hence, there is a protective effect via adipose tissue-VEGF-D-overexpression due to enhanced lymphangiogenesis.

Although human adipose tissue shows noticeable expression of lymphatic vessels (98), very little lymphatics are expressed in murine adipose tissues controversially (95, 97). What are the specific roles of lymphatics in adipose tissues? Which regions of adipose tissues express lymphatics? To further substantiate these findings and questions, future studies utilizing genetically modified mouse models should be used to identify the spatiotemporal and distinct roles of lymphatics in various adipose tissues. In addition, the role of lymphangiogenesis in other metabolic tissues, including liver and skeletal muscle, warrant further investigation.

### Tumorigenesis and Diabetes

Tumor angiogenesis is distinct from other kinds of angiogenesis in terms of timing (99). Angiogenesis is unusually prolonged in some non-malignant processes, however it is still self-limited, such as in pyogenic granuloma or keloid development. Diabetes has been linked to an increased risk of cancer, according to extensive studies (100–102) and increased mortality of cancer patients (103). Previous research on the link between diabetes and cancer has found that diabetics are more prone to develop malignancies of the liver, pancreas, endometrial, colon, rectum, breast, and bladder (104) (Figure 1).

The mechanism of such an increased risk in these patients remains unclear whether: (i) the association is mainly due to shared risk factors such as obesity (105); (ii) diabetes itself alters cancer risk which may be related to insulin resistance (106), hyperinsulinemia (107–109), proinflammatory status and increased oxidative stress (1–35) (110); (iii) the risk of cancer is modified with medications administered to combat diabetes; or (iv) a combination of all these assumptions.

More observational studies have been accumulating with regard to the effects of diabetes treatment in cancer incidence. Insulin growth factor (IGF) is an important hormone for normal and transformed cell growth, development, differentiation and survival, which may play an important role in mammary tumorigenesis and diabetes (111). Several studies have found that anti-hyperglycemic drugs for diabetes treatment may be associated with either an increased or reduced risk of cancer (112, 113). Meta-analysis of observational studies found that while treatments with metformin decrease insulin resistance, it may also reduce the risk of colorectal and hepatocellular cancer in diabetic patients (114, 115), but sulfonylureas and insulin, which may cause hyperinsulinemia, did not show a significant influence. In pancreatic cancer, metformin, thiazolidinediones and insulin use had no significant effect while sulfonylurea use was associated with a 70% increase in the odds of having pancreatic cancer (116). Remaining concerns were expressed for a potential link between pioglitazone (117) and a novel class of oral glucose-lowing drug Sodium-glucose cotransporter 2 (SGLT2) inhibitors (118) with bladder cancer. Besides glucose-lowing drugs, a retrospective study of 92,366 women with newly diagnosed T2D observed a decrease in risk of endometrioid cancer in diabetic patients treated with statins (119). Long-term prospective trials and post-marketing surveillance studies are, however, required in the future.

In addition to increased incidence of cancer, diabetes also has deleterious effects on cancer prognosis. Diabetes was found to be strongly related with an increased risk of death from overall cancer in a study (120) in more than 771,297 Asians with pathologies of the endometrium, liver, thyroid, kidney, breast, ovary, pancreas, and prostate [hazard ratio 1.26 (CI 1.21–1.31)]. Extensive studies have provided the mechanism in which diabetes influences a poor cancer prognosis, including strengthening metastatic potential of cancer, favoring cancer growth (121). Also, impaired immune function in diabetes possibly results in a more aggressive cancer course. At the same time, researchers are trying to use probiotics, especially microbial short-chain fatty acids (SCFAs) to fight against inflammation and protect from tumorigenesis in people with diabetes (122).

Preexisting diabetes is linked to an increased risk of morbidity and mortality in cancer patients, according to all of these studies. There are also studies that investigated the impact of cancer on long term outcomes of diabetes (123). Researchers followed three cohorts of diabetes patients subsequently diagnosed with breast, colorectal or prostate cancer for 10 years, and they found that in the UK, incidence of cancer appears to have little adverse impact on diabetes-related mortality (123).

These findings are clinically meaningful which point to the importance of appropriate cancer screening among diabetic patients and management of diabetic patients with cancer.

### Gut Microbiota Homeostasis and Diabetes

All organisms that live in the gastrointestinal (GI) tract are referred to as gut microbiota. The human body is home to trillions of microorganisms (124), all of which serve a crucial part in normal intestinal function and the host's overall health. Some studies linked gut microbiota with diabetes (Figure 1).

The gut microbiota is mostly formed of four phyla: *Firmicutes, Bacteroidetes, Actinobacteria*, and *Proteobacteria*. Each individual has a unique microbiota composition (125). Diet, disease status, drugs, and host genetics will all have an impact on the composition. Among them, diet is the main contributor to the diversity of microbiota. It has been suggested that diet accounts for 57% of the variations while host genetics only account for 13% (126). Diet has been shown to affect the content and function of the gut microbiome in both animal and human studies. One study (127) switched mice from low-fat, plant polysaccharide–rich diet to a high fat and sugar diet, which altered the composition of the microbiota within a single day. Mice fed a high-fat, high-sugar diet showed a higher number of *Erysipelotrichi* class bacteria in the *Firmicutes phylum* and a lower number of *Bacteroides* spp. (127). *Bacteroides* spp., *E. coli*, and other bacteria were found in reduced numbers in mice on a vegetarian diet (127).

It's becoming clear that gut microbiota has a role in a variety of disorders, including type 1 and type 2 diabetes. T1D is an autoimmune illness caused by the immune system's destruction of pancreatic β-cells. It is mainly caused by a genetic defect as well as epigenetic and environmental factors. Increased rates of T1D incidence in recent years have been attributed to genetic factors as well as changes in lifestyle, such as nutrition, hygiene, and antibiotic use, all of which can have a direct impact on microbiota (128). Several studies have found changes in gut microbiota composition between people with T1D and healthy people. Compared with age matched healthy controls, gut microbiota in children with high genetic risk for T1D showed less diverse and less dynamic microbiota (129). These findings underscore the importance of learning more about the function bacteria may have in the development of T1D (128–131).

Obesity and T2D are also linked to dysbiosis of the gut microbiota, according to extensive research conducted in animal models and humans. Studies in germ-free mice revealed changes in the gut microbiome makeup that could have a role in disease development, including obesity and diabetes (132–134). *AKKermansia muciniphila*, a mucin-degrading bacteria found in the mucus layer, was isolated in one study. In rodents and humans, it has an inverse relationship with body weight. This study found that the abundance of *AKKermansia muciniphila* was reduced in obese and type 2 diabetic mice, and that prebiotic feeding restored *AKKermansia muciniphila* abundance, which was linked to improvements in metabolic diseases such as fat mass gain, adipose tissue and insulin resistance. Butyrate-producing *Roseburia intestinalis* and *Faecalibacterium prausnitzii* concentrations were found to be lower in T2D patients, but *Lactobacillus gasseri* and *Streptococcus mutans*, Proteobacteria, and some Clostridiales were found to be greater among 345 Chinese individuals (135). T2D is also linked to increased bacterial expression of oxidative stress-related genes, resulting in a proinflammatory signature in the gut microbiome (135). All of these studies point to a link between the gut microbiome's makeup and T2D.

For the mechanisms of how gut bacteria affect T2D, most studies focused on the involvement of microbiota in obesity and their role in insulin signaling and low grade inflammation. High-calorie diets contribute to obesity and T2D has been demonstrated by numerous studies (136, 137) and increasing evidence suggests that the link between diet and obesity lies in the gut microbiota (125, 138, 139). One study in mice found that the abundance of *A. muciniphila* decreased in obese and T2D mice. Prebiotic feeding of *A. muciniphila* improved metabolic profiles, and reduced fat mass and insulin resistance induced by high fat diet (140). Qin et al. (135) showed T2D patients had a moderate degree of gut microbial dysbiosis, a decrease in universal butyrate-producing bacteria, which play a role in regulating important T2D pathways including insulin signaling, inflammation and glucosehomeostasis (135, 141, 142). On the other hand, gut microbiota has been shown to affect the production of key insulin signaling molecules such as GLP-1 and PYY through SCFA and its binding to FFAR2 (143). Interestingly, recent studies reported that administration of metformin, the routinely used drug to control hyperglycemia in T2D, alters the composition of the microbiota (144–146).

Numerous studies have suggested that gut microbiota may have a role in the development of diabetes, as well as the importance of gut microbiota in metabolic illnesses that affect key pathways such as energy balance and inflammation. As a result, a better understanding of the relationships between gut microbiota may provide novel therapeutic interventions of diabetes the future.

## Cardiovascular and Other Complications in Diabetes

### Diabetic Retinopathy

**Figure 3:**
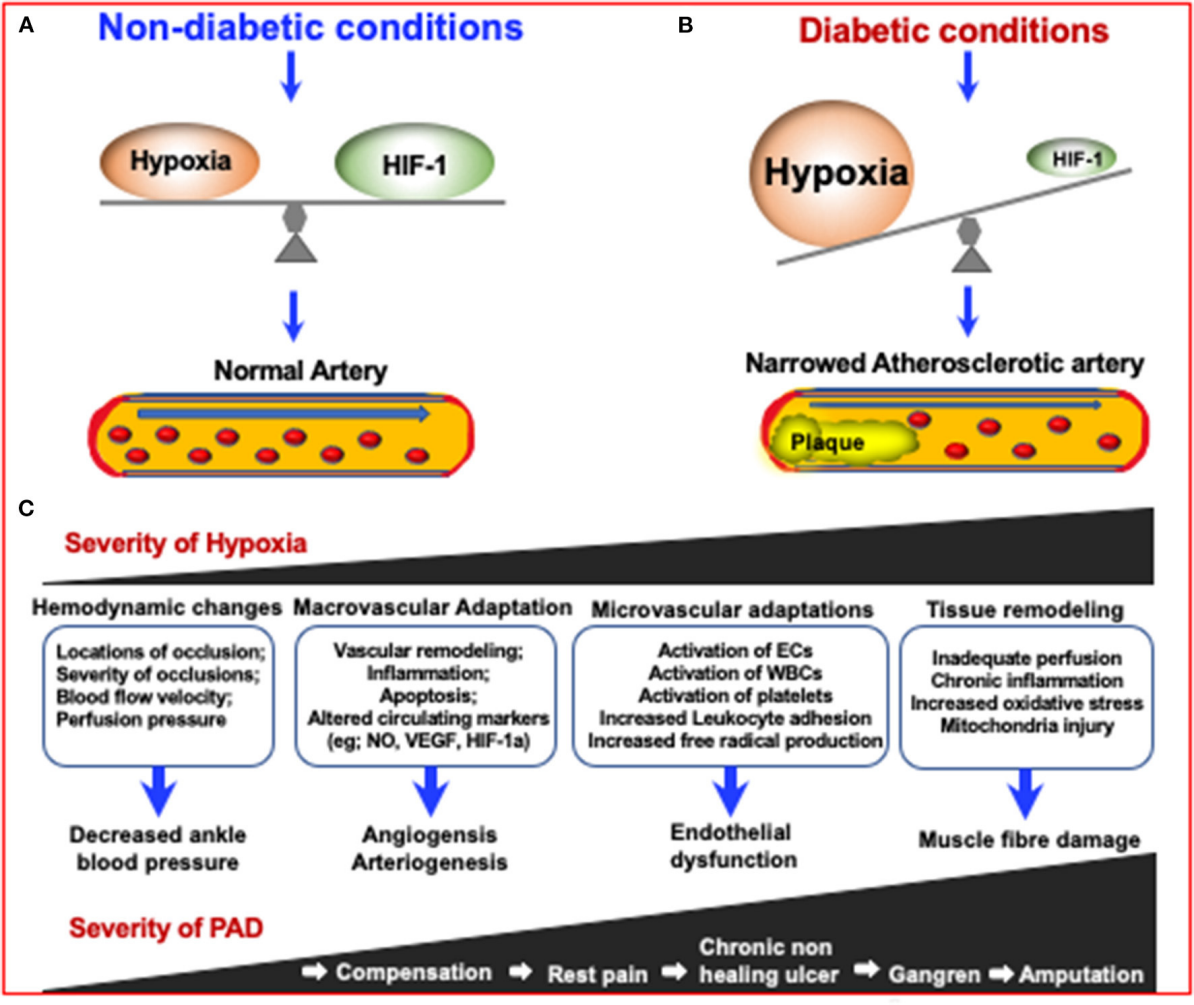
The mechanisms in which diabetes can increase the severity of Peripheral Artery Disease (PAD) *via* increasing the severity of hypoxia which results in narrowing of arteries. Due to the inhibition of HIF-1 in diabetes, an impaired response to hypoxia can lead to diabetes and diabetic complications. **(A)** Under non-diabetic conditions, HIF-1 signaling responds to reduced oxygen levels, resulting in a steady state of hypoxia. **(B)** In the case of diabetes, although the tissue is more hypoxic, HIF-1 signal transduction is inhibited, resulting in impaired adaptive response to hypoxia, leading to the development of diabetes and its complications (155). **(C)** The consequences of increased PAD severity and hypoxia severity are highlighted in the boxes and describe the consequences respective to the degree of PAD severity.

Studies divide diabetic retinopathy (DR) into two progressive stages: non-proliferative retinopathy (NPDR) and proliferative diabetic retinopathy (PDR) (147). Non-proliferative retinopathy is characterized by high glucose which induces dysfunction and structural damage to the retina blood vessels, causing them to leak and dilate (148). At the NPDR stage, vision isn't significantly altered and the condition is asymptomatic (149). PDR on the other hand can result in aberrant, fragile retinal neovessel formation and blindness (150, 151). The vision loss can occur from proliferation of new immature retinal vessels as well as increased leakage and permeability of retinal blood vessels (151). Pericyte loss, which is a hall mark early risk factor for DR, results in local outpouching of capillary walls which is used as a diagnostic for DR (152). Capillary obstruction and ischemia result from a significant loss of pericytes. When pericytes are lost, hypoxia-inducible factor 1 is activated, which causes VEGF to be upregulated (153–155) (Figures 2, 3). Therapeutic drugs such as Pegaptanib, Bevacizumab, Ranibizumab and Aflibercept have been implanted to target VEGF to inhibit its expression since this factor has been found to be highly upregulated in patients with retinopathy (156, 157). These anti-VEGF therapies significantly reduce retinal inflammation, growth of neovessels, and when combined with Ang-2 produces an enhanced effect in reducing retinal inflammation, retinal apoptosis and neovascular leakage (157, 158).

Studies also report that hyperglycemia is a known treatable risk factor for DR. It has been studied that cellular elements in microvasculature are particularly sensitive to damage from hyperglycemia (159). Hyperglycemia is also responsible for apoptosis of pericytes as well as retinal inflammation (160). More importantly however, hyperglycemia plays a broader role in various metabolic pathways, such as upregulated VEGF, increased polyol and PKC pathway activity, chronic oxidative damage, increased activation of renin angiotensin, chronic inflammation and abnormal clumping of leukocytes, which are all involved in the progression of diabetic retinopathy (161–164).

### Diabetic Nephropathy

The thickening of the glomerular basement membrane is a common early change in both type 1 and type 2 diabetic nephropathy, according to studies (165). A related consequence is the expansion of cellular and matrix components in the mesangium which ultimately restricts and distorts glomerular capillaries which diminishes the capillary filtration surface (165). In combination with mesangial expansion, other mechanisms that inhibit glomerular filtration rate include the excess secretion of Semaphorin3a (sema3a) from podocytes (166). Excess sema3a exacerbates other diabetic nephropathy (DN) risk factors such as the development of kimmelstiel-wilson lesions and podocyte effacement and injury (166, 167). Thus, many studies have focused on developing therapeutics to inhibit signaling pathways that promote sema3a such as the JNK and Rac1/NF-κB p65 signaling pathways (167, 168). Oher well known risk factors that are critical to the pathogenesis of DN include deposition of extracellular matrix proteins (ECM), which include collagen, laminin and fibronectin, in the mesangial and the glomerular basement membrane.

Studies have also focused on the ambiguous role of circular RNAs in signaling pathways that result in the promotion of ECMs such as circRNA_010383, circRNA_15698, and circRNA CDR1as/ciRS-7 (169–171). In addition, signaling pathways such as Notch, Wnt, mToR, epac-rpa-1 may all play critical roles in the accumulation of ECMs as well as renal fibrosis (172). Further research should be done on how these signaling pathways are related to ECM accumulation. However, some of those signaling pathways have been shown to have independent roles in DN development and podocyte apoptosis.

For example, in addition to various downstream transcription factors that are thought to regulate the Notch signaling pathway, a recent study has found an additional regulating mechanism via cross talk between miRNAs and the Notch pathway (173). Under high-glucose condition models, overexpression of miR-145-5p inhibited high glucose-induced podocyte cell apoptosis and it was found that the direct target of miR-145-5p was Notch1 (173). Thus, inactivation of the Notch signaling pathway by overexpressing miR-145-5p could attenuate podocyte death in DN. It has also been established that the Wnt pathway plays an independent role in the progression of DN (174). In a study focusing on panax notoginseng (PN), it was concluded that PN plays a role in inhibiting wnt1 in the Wnt/β-catenin signaling pathway which causes a downstream effect of reducing epithelial-mesenchymal transition (EMT), which contributes to podocyte dysfunction, as well as restoring normal protein expression of nephrin (174).

In addition to the critical role of inhibiting Notch and Wnt, inhibition of the mTOR pathway has been studied as a target to ameliorate DN. A recent study found that sperm-associated antigen 5 (SPAG5) plays a role in activating the AKT/mTOR signaling pathway by forming a positive feedback loop with SPAG5-AS1, miR-769-5p and transcriptional repressor YY1 (175). Furthermore, it was concluded that regulating expression of SPAG5 could regulate podocyte injury under high glucose conditions since SPAG5 directly regulates the AKT/mTOR pathway (175). Another study demonstrated that transplantation of adipose-derived stem cells (ADSC) derived from exosome (ADSC-exo), attenuated podocyte damage in DN (176). The mechanism being that ADSCs-exo mediates the transport of miR-486 to podocytes by regulating activation of the mTOR pathway, leading to decreased podocyte injury (176).

Other critical pathways that lead to podocyte injury and dysfunction involve KDM6A and KLF10 which present a positive feedback loop in podocytes causing podocyte dysfunction under diabetic conditions (177). This pathway is so critical that mouse models were protected against diabetic induced treatment once this pathway was inactivated (177). Other pathologies that lead to podocyte dysfunction include nephrin down-regulation (178). Involved in nephrin downregulation is PACSIN2 which has been found to be highly expressed in podocytes of diabetic animal models (178). Though the relationship between PACSIN2 and nephrin is till speculative, studies have shown that nephrin relies on a complex of PACSIN2 and rabenosyn-5 for nephrin endocytosis and recycling (178, 179). Thus, overexpression of PACSIN2 combined with rabenosyn-5 could increase nephrin endocytosis resulting in a breakdown of podocyte effacement, ultimately leading to podocyte dysfunction and death.

Cell senescence could be another mechanism in which DN occurs according to some studies. Conversely, the disease conditions presented by DN also most likely accelerate the progression of the disease (180). Cell senescence has been found to cause a loss of self-repair in cells as well as their regenerative ability (180). This would be particularly pathological to renal cells leading to accelerated aging of the kidney. Hyperglycemia in diabetic patients is related to the production of reactive oxygen species (ROS) that cause oxidative stress resulting in activation of pathways which cause renal damage and onset cell senescence (181). Similarly, studies have found that overproduction of mitochondiral reactive oxygen species (mtROS) in DN due to excessive metabolic demand could also be mechanism that could lead to damaged renal cells (182).

Diabetic nephropathy is a progressive kidney disease affecting kidney glomeruli, arterioles, tubules and the interstitium. Dapagliflozin and Prevention of Adverse outcomes in Chronic Kidney Disease (DAPA-CKD), a randomized controlled trial (183, 184), demonstrated beneficial kidney and cardiovascular outcomes with dapagliflozin vs. placebo in participants with Chronic Kidney Disease (CKD) with and without diabetes (185). More prespecified analyses from this landmark trial have confirmed dapagliflozin's cardio-renal protective effects, in favor of combined therapy (186). In light of the mechanistic findings presented in this review, future studies should test integrative approaches to identify dual or multiple targets to deal with this complex disease.

### Diabetic Cardiomyopathy

There is a significant relationship between the prevalence of heart failure and diabetes. In the absence of other traditional cardiac risk factors such as coronary artery disease, hypertension, and valvular heart disease, diabetes alone can cause heart failure, namely diabetic cardiomyopathy (DCM), presenting pathological changes in cardiac structure, metabolism, and function (187). In fact, diabetes is prevalent in anywhere between 10 and 40% of heart failure subjects due to cardiomyopathy (188). The major manifestations include cardiac stiffness, myocardial fibrosis, and hypertrophy, eventually progressing to clinical heart failure (189). Due to its' significant impacts on human health, the mechanisms behind the pathogenesis of DCM have been a hot topic of research.

The pathological factors of diabetes relevant to the pathogenesis of cardiomyopathy include hyperlipidemia, hyperglycemia and systemic insulin resistance (190). Hyperlipidemia and hyperglycemia were discussed in a recent study in their relationship to inhibiting expression of transcription factor Sp1 which was shown to be involved in downregulating mitochondrial calcium uptake 1 (MICUI1) (191). More specifically, restoring normal function of mitochondrial calcium uptake 1 (MICU1), which was confirmed to be downregulated in the hearts of diabetic mice via hyperlipidemia and hyperglycemia, was important for inhibiting the progression of cardiomyopathy (191). Thus, confirming with previous studies, it was concluded that reduced mitochondrial Ca^2+^ uptake *via* downregulated MICU1 caused dysfunction in diabetic hearts (191). Hyperglycemia and hyperlipidemia also play crucial pathological roles in cardiomyopathy via hyperglycemia-induced oxidative stress and fibrosis development due to increased ROS generation (192). Sirtuin 1 (SIRT1) is a deacetylase that has been previously shown to have a protective effect against cardiovascular disease in the context of resisting sustained oxidative stress (193). Thus, it would be very therapeutically beneficial to target SIRT1 as a means of reducing or preventing cardiomyopathy. This rational was implemented in this study focusing on the role of Tetrahydrocannabinol (THC) in mitigating oxidative stress caused by hyperglycemia by activating SIRT1 (192). The study confirmed with past research that SIRT1 is inhibited due to high glucose levels in the mouse hearts of diabetic cardiomyopathy models. The study also confirmed that superoxide Dismutase 2 (SOD2) is a product of SIRT1 activation and plays the main role in regulating ROS homeostasis when deacetylated. Interestingly, SIRT1 was dramatically upregulated when THC was administered and any pathological downstream transformations, such as reduction of deacetylation SOD2, were shown to be reversed (192). This is a novel therapeutic finding that hasn't been demonstrated before. It is also worth noting that activation of SIRT3 combined with administration of melatonin had a similar protective effect in reducing oxidative stress brought on by hyperglycemia (194). However, SIRT4 did not have these same protective effects and in fact was found to promote cardiac dysfunction by increasing ROS levels (195).

Although hyperglycemia, systemic insulin resistance, and hyperinsulinemia are regarded as the key etiological factors of DCM (187), multiple mechanisms may act at systemic, myocardial, and cellular/molecular levels, including metabolic abnormalities (e.g., lipotoxicity and glucotoxicity), mitochondrial damage and dysfunction, oxidative stress, abnormal calcium signaling, inflammation and epigenetic factors. For example, recent studies demonstrated in diabetic animal models that decreased cardiomyocyte function is a potential mechanism leading to DCM, which could result from decreased AMPK signaling, or increased AMP-activated protein kinase (MPK) signaling and increased protein kinase C (PKC) and mitogen-activated protein kinase (MAPK) signaling. Upregulation of double-stranded RNA-activated protein kinase (PKR) pathway also caused glucolipotoxicity in DCM (196). A new study using multi-omics technology in a HFD-STZ model showed that the formation of short-chain acylcarnitine species in T2D mouse hearts activated networks to redistribute excess acetyl-CoA toward ketogenesis and incomplete β-oxidation, resulting in loss of metabolic flexibility and the capacity of the heart to respond to subsequent cardiovascular events (197). Clearly, these disturbances would predispose the heart to extracellular remodeling and hypertrophy, both eventually leading to heart failure (187). Along with the deleterious hyperglycemic and hyperlipidemic effects on DCM, immerging research has analyzed the impact of HFD-induced diabetes on cardiac dysfunction in the context of lipotoxicity (198). The transcription factor studied was PPAR-γ, which has been demonstrated to regulate the expression of genes related to lipid metabolism (198). Consistent with previous studies, PPAR-γ was found to be highly expressed in diabetic heart models, however this study first demonstrated that PPAR-γ was directly associated with upregulation of ketogenic enzymes HMGCS2, PDK4 and BDH1 all of which are involved in controlling lipotoxicity and subsequent cardiac dysfunction (198–200). Therefore, ablation of PPAR-γ in diabetic-heart mouse models lead to improvements in cardiac contraction and prevention of fibrosis development both of which are suggestive of better cardiac function (198).

Some research has been done on the expression of non-coding RNAs (LncRNAs) and their involvement in the pathogenesis of DCM. To highlight a single study, it was found that LncRNA Kcnq1ot1 was significantly upregulated in high glucose cardiac fibroblasts as well as diabetic myocardial tissues (201). The main pathological pathway discussed in relation to DCM involved regulating caspase-1, the hypothesis being that downregulating Kcnq1ot1 repressed activation of miR-214-3p which reduced expression of caspase-1 and its downstream inflammatory cytokines such as interleukin 6, interleukin 10, and the IL-1 family, all of which are involved in DCM induced heart failure (202). Other studies that focused on lncRNAs, such as myocardial infarction–associated transcript (MIAT) (203), myosin heavy-chain-associated RNA transcripts (Mhrt) (204), and H19/miR-675 (205), all similarly found that forced expression or overexpression of these lncRNAs lead to preservation of cardiomyocyte apoptosis involved in the pathogenesis of DCM. Thus, there is therapeutic protentional in knocking out these specific lncRNAs in ameliorating DCM.

Taken together, it is clear that there is emerging research in the area of therapeutically treating DCM. As described above, numerous findings have been made that deal with the mechanisms behind DCM, such as targeting certain transcription factors like Sp1, deacetylases such as SIRT1 and oxidative stress (206). There are also some findings dealing with blocking long non-coding RNA (207) like Kcnq1ot1, Mhrt, and exsosomal miRNAs, like H19/miR-675 all of which have been demonstrated to promote DCM (208). Future studies should show additional mechanistic findings through the use of advanced and integrative methodologies.

### Diabetic Complications in Peripheral Artery Disease

Peripheral artery disease (PAD) by itself affects 27 million individuals in both Europe and North America annually (209). The known risk factors of PAD are old age, risk of cardiovascular disease, and ethnicity, specifically if one is of Hispanic or African American descent (210). Diabetes in relation to PAD has been studied and has been shown to exacerbate PAD. For example, a 20 year follow up study found a significant increased risk of death for patients with diabetes and PAD compared to patients without diabetes (211). Thus, research in how diabetes contributes as a risk factor for PAD-associated mortality is critical (Figure 3).

The main mechanisms in which diabetes fosters the development of PAD are mainly through the same mechanisms that cause cardiovascular disease. Derangements in the vessel wall caused by vascular inflammation and endothelial cell dysfunction, aberrant blood cells, and an increase in reactive oxygen species are among these mechanisms (155, 212) (Figure 3). Hyperglycemia, a pathological consequence of diabetes, causes damage to the vascular endothelium in a variety of pathways such as the protein kinase C and advanced glycation end products pathways all of which lead to dysregulation of growth factors, cytokines, epigenetic changes, and abnormality of non-coding RNAs leading to macrovascular complications such as PAD (213). Another way in which hyperglycemia can cause damage to the vascular system is by inducing hypoxia which leads to oxidative stress (OxS) and subsequent vascular damage (214). This mechanism is illustrated in Figure 3. Moreover, oxidative stress (OxS) seems to play a pathophysiological role in PAD and atherosclerosis via its association with the production of reactive oxygen species ROS. Production of ROS from OxS is caused by OxS impairing nitric oxide (NO) synthesis (215). Over production of ROS, which at low levels act as signaling molecules that mediate vascular cell proliferation, migration, and differentiation, can be detrimental to microvascular angiogenesis (216). Therefore, it is clear there is some sort of cascading affect between OxS and cardiovascular diseases such as PAD, and oxidative stress biomarkers are key for identifying their progression (215).

However, there are some studies that speculate alternative mechanisms in which diabetes causes PAD. For example, one study found that serum levels of omentin-1, an adipocytokine, were significantly lower in diabetic patients with PAD compared to diabetic controls without PAD and that these levels significantly dropped as disease severity progressed (217). This is the first study to assess reduced serum omentin-1 levels as being a potential biomarker for PAD and thus should be researched more (217). Hyperglycemia was reported to hyperphosphorylated PKCβ in diabetic animal models resulting in impaired ischemia-induced activation of the canonical NF-κβ signaling pathway and inferior experimental PAD outcomes (218). Interestingly, both omentin-1 and PKCβ expression levels had significant correlations with severity of PAD in diabetic models. Another potential useful biomarker that was studied included prolonged heart rate-corrected QT interval (QTc) which was found to have a significant positive association with severity of PAD in patients that have diabetic foot ulcers (219). Additionally, there is some evidence that links elevated leptin levels with the presence and severity of PAD and diabetes (220).

To further highlight potential therapeutic targets, one study concluded that glucose normalization could be targeted as a therapeutic in diabetic patients to ameliorate PAD (221). Specifically, this study showed that impaired VEGFR expression, via greater ubiquitination under high glucose conditions, was linked to impaired perfusion recovery in Type 1 diabetes. Another study focused on acylated ghrelin (AG) and found that plasma AG was significantly lower in animal models with diabetes and PAD (222). By modulating specific miRNAs such as miRNA 126 and 132, they were able to influence the expression levels of AG which promoted a proangiogenic response. In other words, restoring AG to normal plasma levels lead to revascularization in diabetic and PAD models which means that exogenous AG could be a promising therapeutic for treating PAD in diabetic patients (222).

Micro RNAs (miRs) seem to play an independent role in diabetic PAD; however, more research needs to be done on the specific mRNAs that affect diabetic PAD. To exemplify a few, diabetes-induced upregulation of mRNA-133a was found to impair angiogenesis in PAD by reducing nitrogen oxide synthesis in endothelial cells (223). Conversely, inhibiting expression of miR-133a resulted in improved angiogenesis after experimental post-ischemic inducement in diabetic mice (223). Another miR identified as miR-93 enhanced blood perfusion after experimental hind limb ischemia, making it a valuable target for modulation in promoting angiogenesis particularly in ischemic tissue (224). Thus, there is some evidence to support that miRs play an important role in recovering from PAD reduced blood perfusion; however, more investigation is warranted.

Studies have shown that diabetes accelerates atherosclerosis and that peripheral artery disease is a marker of advanced atherosclerosis (225). Patients with PAD and diabetes were found to have an increased risk of cardiovascular death or ischemic stroke as well as a higher risk for lower extremity amputations compared to patients with PAD and no diabetes (226). More specifically, patients with T2D and PAD had lower heart rate variability which is indicative of autonomic dysfunction (227). Furthermore, autonomic cardiovascular dysfunction in relationship with atherosclerosis has been studied. Specifically it was found that diabetic patients with low heart rate variability had higher levels of inflammatory markers such carotid intima-media thickness (IMT) (228).

Concerning the lower extremity amputation risk, advanced PAD can result in chronic limb threatening ischemia (CLTI) which is associated with a higher risk of lower limb loss (229). Since amputation risk of lower extremities is a common complication of patients with PAD and diabetes (230), it is important to be able to assess PAD risk among diabetic patients before they develop diabetic foot ulcers in which amputation then becomes more necessary. One major indicator of amputation risk that can be non-invasively assessed is by determining the ankle brachial index (231). The results of this study showed that patients who exhibited a lower ankle brachial index were at a higher risk for a foot ulcer and thus an amputation.

An emerging method that could be more predictive include ultrasound measurement methods that, for example, can be used in plantar soft tissues in order to predict diabetic related changes in the foot (232). In one study, it was found that Sub-MTH fat pads were significantly thinner and subhalangeal fat pads were significantly thicker in diabetic neuropathic feet compared to neuropathic controls (233). Another study conducted in China used similar ultrasonographic methods to detect foot muscle atrophy in Chinese patients with type 2 diabetes mellitus. The extensor digitorum brevis muscles (EDB) as well as the muscles of the first interstitium (MILs) had a reduced transverse diameter, thickness, and cross sectional area in all of the patients' nondominant feet, according to this study (234). In other studies, it has been found that the use of ultrasound can assess a significant reduction in the thickness of the intrinsic foot muscles and plantar tissues in patients with T2D (235). There is also evidence that people with T2D have stiffer heel pads (236). Taken together, these studies represent useful data for using ultrasonography as a noninvasive and cost effective way to detect early diabetic complications in the foot. Advancement in this technology would be critical for patients who have diabetes and PAD in order to prevent or reduce their need for amputation (Figure 3).

## Perspectives

Currently the incidence and prevalence of diabetes mellitus around the world is very high and diabetes has become a threat to mankind globally. With the advancement of genomics, epigenomics, proteomics, and multiomics single-cell analyses, more promising and powerful approaches for mechanistic studies of diabetes have come to fruition. Also, the association between diabetes and other physiological systems, especially the cardiovascular system, revealed more potential pathways and targets involved in the progression of diabetes. Therapeutically intervening in these pathways will also help us to mitigate the effects that diabetes has on other pathologies described in this review such as retinopathy, nephropathy, peripheral artery disease, and cardiomyopathy. The information described in this paper would present a tremendous leap forward in predicting, diagnosing, managing and treating diabetes.

## Author Contributions

HW, VN, and HC proposed the conception. HW, VN, KC, BZ, SB, YWL, BW, DS, SW, S-LC, YD, DC, JX, DRB, CZ, and HC performed the literature search complied and wrote the manuscript. HW, VN, and HC proofed the manuscript and figures. All authors contributed to manuscript draft and revision.

## Funding

This work was supported in part by NIH Grants R01HL093242, R01HL130845, R01HL133216, R01HL137229, R01HL156362, and R01HL158097 and American Heart Association Established Investigator Award to HC, R01HL141858 to DRB, NIH R01ES023470, NIH R01HL131925 to CZ, and Scientist Development Grant 17SDG33410868 and NIH T32 to HW.

## Conflict of Interest

The authors declare that the research was conducted in the absence of any commercial or financial relationships that could be construed as a potential conflict of interest.

## Publisher's Note

All claims expressed in this article are solely those of the authors and do not necessarily represent those of their affiliated organizations, or those of the publisher, the editors and the reviewers. Any product that may be evaluated in this article, or claim that may be made by its manufacturer, is not guaranteed or endorsed by the publisher.
